# Purulent Pericarditis and Mycotic Pseudoaneurysm of the Ascending Aorta: A Case Report

**DOI:** 10.7759/cureus.72857

**Published:** 2024-11-01

**Authors:** Oswaldo Subillaga, Mohamed S Omer, Neel R Sodha

**Affiliations:** 1 Department of Surgery, The Warren Alpert Medical School, Brown University, Providence, USA; 2 Department of Pathology and Laboratory Medicine, The Warren Alpert Medical School, Brown University, Providence, USA

**Keywords:** ascending aorta aneurysm, bovine pericardial patch, mycotic pseudoaneurysm, purulent pericarditis, staph-aureus

## Abstract

Concomitant presentation of purulent pericarditis and mycotic pseudoaneurysm of the ascending aorta is exceedingly uncommon. We present a case of a 63-year-old male who presented to the emergency department after one week of severe neck pain along with pleuritic chest pain. He was found to have purulent pericarditis associated with a 0.9cm pseudoaneurysm of the ascending aorta. He underwent surgical repair with a bovine pericardial patch as well as mediastinal debridement, which revealed a larger area of necrosis than anticipated based on imaging. Microbiological investigation revealed the presence of Methicillin-sensitive Staphylococcus aureus (MSSA) in both tissues from the aortic pseudoaneurysm and the pericardial rind.

## Introduction

Purulent pericarditis and mycotic pseudoaneurysms of the ascending aorta are both individually rare and often fatal. Concomitant presentation of these two pathologic processes is exceedingly uncommon, with only about a dozen cases involving the thoracic aorta reported in the literature [1-2].

Before the widespread use of antibiotics, the incidence of purulent pericarditis was estimated to be 1 in 254 persons, accounting for almost half of all cases of pericarditis [3]. In contrast, the most recent estimates calculate its incidence to be 1 in 18,000 persons [3]. The presentation is typically acute and is characterized by nonspecific symptoms such as fever, tachycardia, cough, leukocytosis, and chest pain. It is most often seen in immunocompromised patients and either following trauma to the chest or surgery [2]. Staphylococcus and Streptococcus species are the most common causative pathogens [1,4]. Despite treatment, close to half of patients die from either tamponade, constriction, or disseminated infection [5]. Because death is inevitable without any treatment, immediate action is required following high clinical suspicion of purulent pericarditis.

Mycotic pseudoaneurysms are bacterial or fungal endarteritis causing erosion of endothelial layers and an outpouching contiguous with the arterial lumen. They can be caused by direct inoculation during surgery or trauma, hematogenous spread, or contiguous infection [6]. They are much more frequently seen in the abdominal aorta, femoral artery, or carotid arteries and are most often associated with Salmonella and Staphylococcus infection [2,7-8]. They may also present with nonspecific symptoms [9]. Despite representing less than 2% of all aortic aneurysms, mycotic aneurysms carry high operative mortality (10%-40%) and morbidity (60%-70%) [2, 10-11]. Because of the high rate of morbidity and mortality, high clinical suspicion and early detection are essential as early treatment may improve long-term survival.

We report a case of a 63-year-old gentleman who was found to have purulent pericarditis associated with a mycotic pseudoaneurysm of the ascending aorta.

## Case presentation

A 63-year-old male presented to the emergency department complaining of approximately one week of severe neck pain along with pleuritic chest pain that radiated to the interscapular area and anterior chest. His medical history was significant for migraine headaches, smoking, and chronic neck pain with recent cortisone injections and chiropractic manipulation. On presentation, the patient was afebrile, but tachycardic up to 122 beats per minute. He was hypertensive and saturating well without supplemental oxygen. He was found to have muffled heart sounds and mild jugular venous distention on the exam.

**Figure 1:**
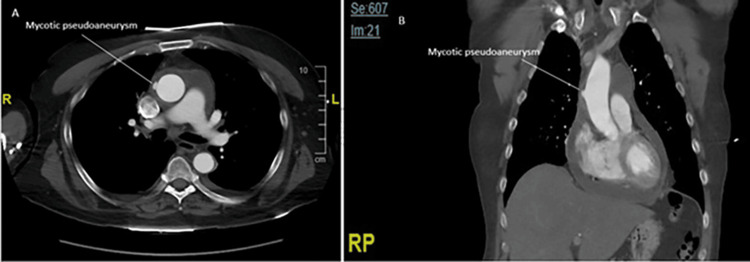
Preoperative computed tomography (CT) angiography of the chest. (A) Axial and (B) coronal contrast-enhanced CT imaging demonstrating the mycotic pseudoaneurysm at the time of presentation.

Blood work revealed leukocytosis and an elevated anion gap metabolic acidosis (Table 1). His international normalized ratio, C-reactive protein, and erythrocyte sedimentation rate were also elevated. Computed tomography angiography (CTA) revealed a 9 mm pseudoaneurysm, approximately 5.5 cm distal to the aortic valve, and a large pericardial effusion (Figure 1). A transthoracic echocardiogram was obtained upon admission and showed normal biventricular function with the known pericardial effusion but without any evidence of valvular disease or vegetation. Blood cultures were obtained but were pending before intervention, and he was started on broad-spectrum antibiotics. A pericardiocentesis yielded 150 cc of serosanguinous fluid, which, upon cytological microscopic examination, revealed acute inflammation. This was followed by a gram stain, indicating the presence of gram-positive cocci. Because of the active infection and risk of possible rupture, surgical repair was planned for the next morning.

**Table 1:** Laboratory values at presentation. ^*^Abnormal value.

Lab value	Patient	Reference range
White blood cell count	23,600 cells/μL^*^	4,500-11,000 cells/μL
Hemoglobin	13.3 g/dL	13-18 g/dL
Hematocrit	39.3%^*^	40%-54%
Platelets	365,000 μL	150,000-400,000 μL
Sodium	127 mEq/L^*^	135-145 mEq/L
Potassium	5.0 mEq/L	3.7-5.2 mEq/L
Chloride	95 mEq/L^*^	96-106 mEq/L
Carbon dioxide	18 mEq/L^*^	23-29 mEq/L
Blood urea nitrogen	22 mg/dL	6-20 mg/dL
Creatinine	0.87 mg/dL	0.6-1.3 mg/dL
Glucose	139 mg/dL^*^	70-100 mg/dL
Calcium	9.2 mg/dL	8.5-10.2 mg/dL
Anion Gap	14 mEq/L^*^	4-12 mEq/L
Erythrocyte sedimentation rate	130 mm/hour^*^	≤20 mm/hour
C-reactive protein	387.35 mg/dL^*^	<0.3 mg/dL

**Figure 2:**
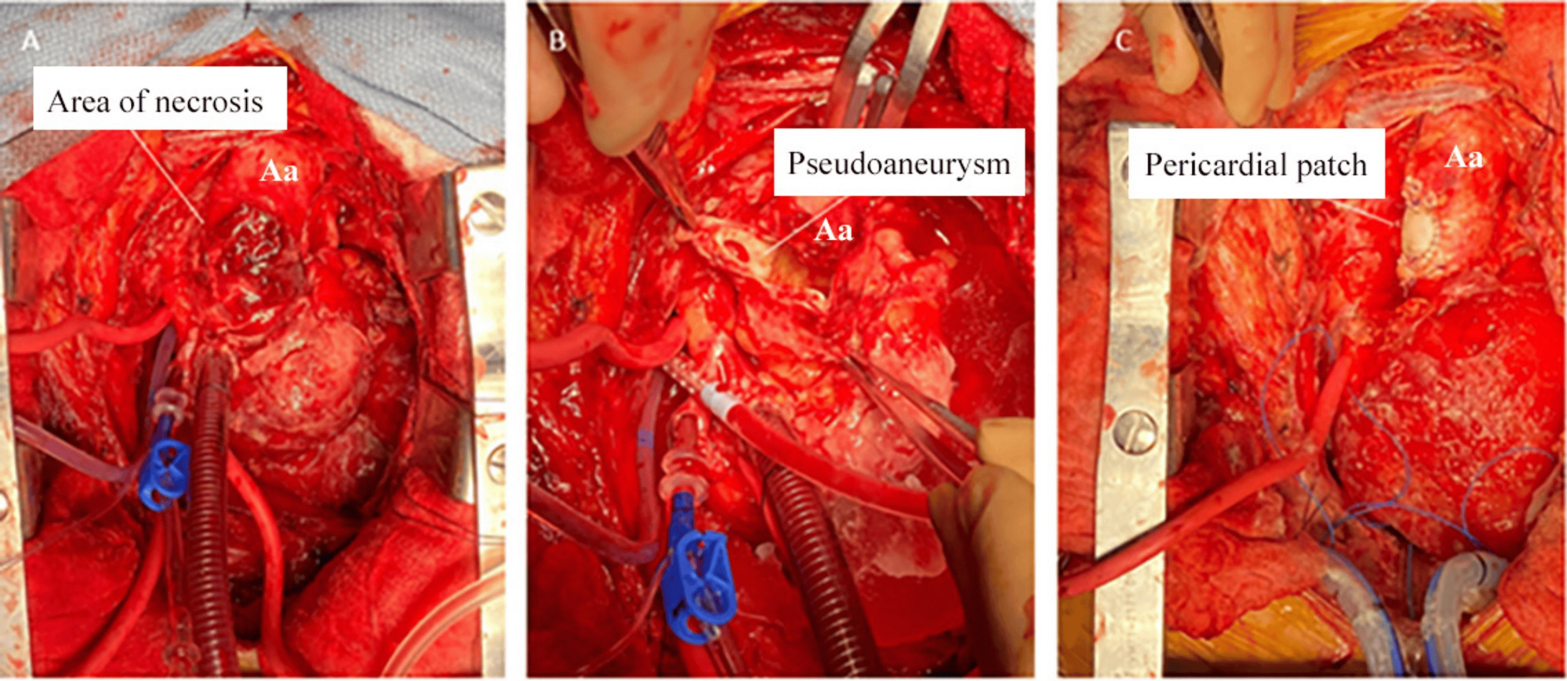
Intraoperative images. Intraoperative images showing (A) the external appearance of the pseudoaneurysm, (B) the internal view, and (C) immediately following patch angioplasty. Aa, ascending aorta

**Figure 3:**
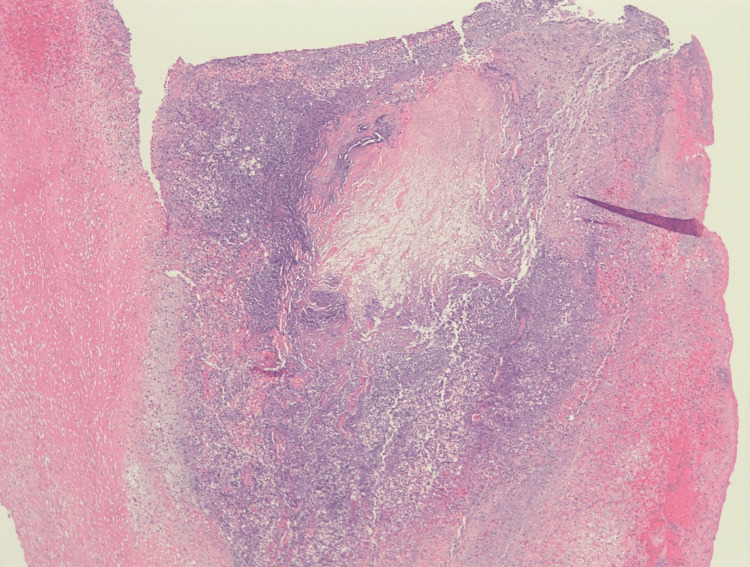
Hematoxylin and eosin-stained aortic pseudoaneurysm (40x), extensive necrosis, fibrin deposition, granulation tissue, and abscess formation with numerous bacteria seen.

**Figure 4:**
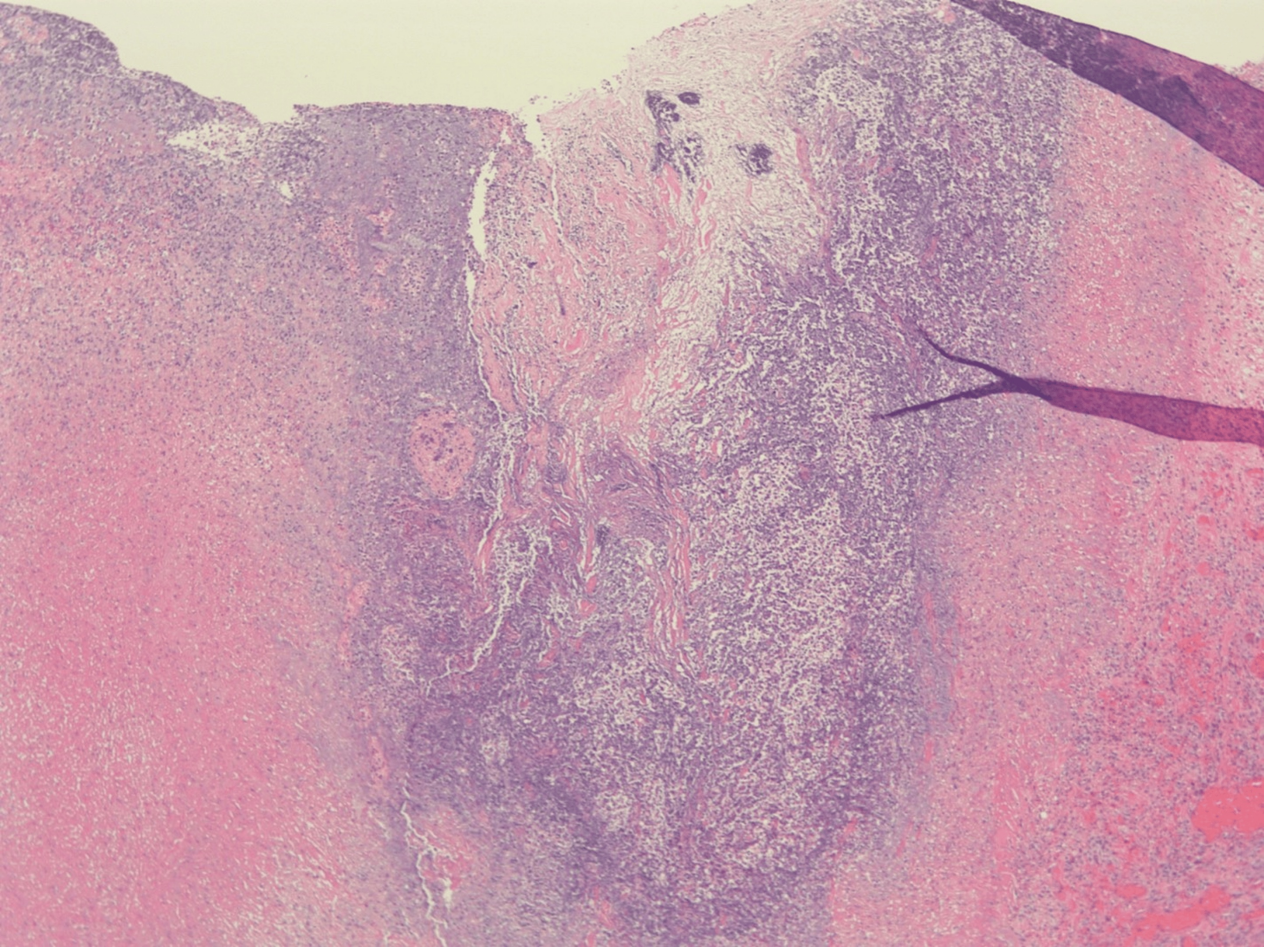
Hematoxylin and eosin-stained pericardial rind (40x) showing granulation tissue and organizing fibrin, focally incorporating many neutrophils.

A bovine pericardial patch repair of the ascending aortic pseudoaneurysm and mediastinal debridement was performed (Figure 2). His pericardium was very thickened, and there was a dense rind of inflammatory tissues around the heart along with 150 cc of residual pericardial effusion. The aortic pseudoaneurysm in the mid-ascending aorta had a neck of approximately 1 cm and necrotic tissues that extended radially from the base of the pseudoaneurysm for approximately 1.5 cm in all directions. After debriding away all the tissue to the healthy aorta, there was an approximately 3.5 cm x 3 cm defect on the anterolateral surface of the aorta that was repaired with a bovine pericardial patch. This area of the necrotic aorta was significantly larger than the size of the visualized pseudoaneurysm. Bovine pericardial pledgets were utilized instead of the usual synthetic ones to avoid a chronic infection and the possibility of rupture if a foreign body is used in an infected field. Tissue from both the aortic pseudoaneurysm and pericardial rind grew Methicillin-sensitive Staphylococcus aureus (MSSA). Histopathologic analysis of the pseudoaneurysm showed extensive necrosis in the pseudoaneurysm with abscess formation (Figure 3). Histopathologic analysis of the pericardial rind showed granulation tissue with many neutrophils present (Figure 4).

**Figure 5:**
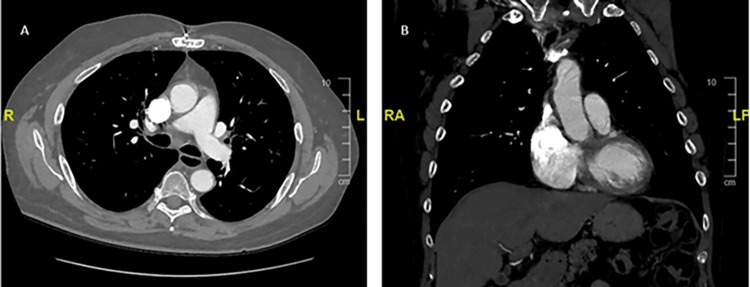
Postoperative computed tomography (CT) angiography. (A) Axial and (B) coronal contrast-enhanced CT imaging four months postoperatively.

Initially admitted to the cardiothoracic intensive care unit, he ultimately recovered and was discharged a couple of weeks following the repair. A CTA obtained four months postoperatively showed no evidence of aneurysm, dissection, or infection (Figure 5).

## Discussion

Although exceedingly rare, the concomitant presentation of purulent pericarditis with a mycotic pseudoaneurysm of the ascending aorta could be catastrophic. Meier et al. reported one such case and compiled 11 prior cases reporting concomitant purulent pericarditis with mycotic aneurysms or pseudoaneurysms of the thoracic aorta going back to the 1960s [2]. While not all the cases involved the ascending aorta, they nonetheless provide important context for our case. Like our patient, most of the ones previously reported were in the seventh decade of life. Some had comorbidities rendering them as immunocompromised. Staphylococcus and Streptococcus species were found in most cases. Of those treated non-operatively, 33.3% (2/6) survived, while 85.7% (6/7) of those treated surgically survived, including our patient [2]. This suggests that surgical repair may provide a better chance of survival.

The rate of growth and rupture is unknown in the setting of a mycotic pseudoaneurysm due to the diagnostic challenges that it poses [12]. However, there are reports showing aneurysm growth in as little as five to 10 days, supporting the role of early surgery in the presence of high clinical suspicion [2,4-5]. Further, our case shows that though they may appear small and relatively benign on imaging, the underlying tissue necrosis may extend beyond the visible pseudoaneurysm. While imaging studies for our patient showed a pseudoaneurysm measuring 0.9 cm, intraoperatively, we encountered an area of necrosis measuring 3.5 cm x 3 cm. Thus, it is essential to consider earlier surgical intervention to prevent further growth or a catastrophic rupture.

Interestingly, our patient, like most others with a similar presentation, was found not to have structural abnormalities or valvular pathology. Our patient also did not have signs of infective endocarditis. In some cases, purulent pericarditis seemed to be the primary problem that was then complicated by a mycotic aneurysm of the aorta. For our patient, however, the distinction between the primary pathology and the complications was not clear. It is thus essential to have a high clinical suspicion for this potentially deadly combination in the appropriate clinical setting.

## Conclusions

The rate of growth or rupture is unknown in the setting of concomitant purulent pericarditis and mycotic pseudoaneurysm of the ascending aorta. Both entities independently convey significant morbidity and mortality. Although a mycotic pseudoaneurysm may appear small and relatively benign on imaging, the underlying tissue necrosis may extend beyond the visible pseudoaneurysm. Thus, it is essential to have a high index of suspicion and proceed with surgical repair expeditiously.
